# A Hybrid Millimeter-Wave Radar–Ultrasonic Fusion System for Robust Human Activity Recognition with Attention-Enhanced Deep Learning

**DOI:** 10.3390/s26031057

**Published:** 2026-02-06

**Authors:** Liping Yao, Kwok L. Chung, Luxin Tang, Tao Ye, Shiquan Wang, Pingchuan Xu, Yuhao Bi, Yaowen Wu

**Affiliations:** 1 School of Intelligent Manufacturing and Electrical Engineering, Guangzhou Institute of Science and Technology, Guangzhou 510540, China; yaoliping@gzist.edu.cn (L.Y.); yetao@gzist.edu.cn (T.Y.); pingchuan.xu@gzist.edu.cn (P.X.); biyh_guet@163.com (Y.B.); wuyaow@gzist.edu.cn (Y.W.); 2 Guangdong Industrial Robot Integration and Application Engineering Technology Research Center, Guangzhou 510540, China; tangluxin@gzist.edu.cn; 3 School of Electrical and Electronic Engineering, Nanyang Technological University, Singapore 639798, Singapore

**Keywords:** millimeter-wave radar, ultrasonic sensor, wavelet transform, short-time Fourier transform, human behavior recognition

## Abstract

**Highlights:**

**What are the main findings?**

- Fusing 77 GHz millimeter-wave radar and 40 kHz ultrasonic signals (with wavelet transform for radar and STFT for ultrasound) overcomes the range-vs-accuracy tradeoff of single-sensor systems, enabling 98.6% accurate recognition of four core human behaviors (standing, sitting, walking, falling) in a privacy-preserving, lighting-agnostic manner.
- The proposed Attention-CNN-BiLSTM architecture—integrating CNN (local spatial features), BiLSTM (bidirectional temporal dependencies), and attention (salient cue enhancement)—outperforms single-sensor baselines and traditional deep learning models, providing a robust technical solution for contactless human behavior recognition.

**What are the implications of the main findings?**

- The mmWave radar-ultrasonic fusion paradigm provides a privacy-preserving, environment-robust solution for contactless human behavior recognition, with direct implications for advancing smart home monitoring, elderly healthcare, and privacy-sensitive surveillance systems.
- The integration of targeted time–frequency feature extraction (wavelet/STFT) and the Attention-CNN-BiLSTM architecture offers a scalable methodological framework for addressing the range-vs-accuracy tradeoff in multi-modal sensing, informing future research on contactless activity recognition.

**Abstract:**

To address the tradeoff between environmental robustness and fine-grained accuracy in single-sensor human behavior recognition, this paper proposes a non-contact system fusing 77 GHz SIFT (mmWave) radar and a 40 kHz ultrasonic array. The system leverages radar’s long-range penetration and low-visibility adaptability, paired with ultrasound’s centimeter-level short-range precision and electromagnetic clutter immunity. A synchronized data acquisition platform ensures multi-modal signal consistency, while wavelet transform (for radar) and STFT (for ultrasound) extract complementary time–frequency features. The proposed Attention-CNN-BiLSTM architecture integrates local spatial feature extraction, bidirectional temporal dependency modeling, and salient cue enhancement. Experimental results on 1600 synchronized sequences (four behaviors: standing, sitting, walking, falling) show a 98.6% mean class accuracy with subject-wise generalization, outperforming single-sensor baselines and traditional deep learning models. As a privacy-preserving, lighting-agnostic solution, it offers promising applications in smart homes, healthcare monitoring, and intelligent surveillance, providing a robust technical foundation for contactless behavior recognition.

## 1. Introduction

Human behavior recognition underpins intelligent surveillance, healthcare, and smart-home systems, yet video-centric approaches increasingly falter under privacy constraints, variable illumination, and occlusion [1,2,3]. Contactless sensing—particularly millimeter-wave (mmWave) radar and ultrasound—has therefore emerged as a privacy-preserving, lighting-agnostic alternative [4,5,6]. mmWave radar penetrates fabrics and operates reliably in darkness, smoke, or heavy rain, while micro-Doppler signatures reveal gait, sitting, standing, or falls [7,8,9]. Its sensitivity to clutter, multipath, and electromagnetic noise, however, limits fine-grained discrimination [10,11]. Ultrasound, conversely, provides centimeter-level accuracy at close range, is inexpensive, and is robust to non-target reflections [12,13]. Fusing the two modalities, therefore, offers a natural path to combine long-range robustness with short-range precision.

Despite this potential, single-sensor schemes still trade environmental resilience for accuracy, whereas existing multi-sensor studies either concatenate raw data or perform late-score fusion without modeling the complementary time–frequency structure of radar and ultrasonic echoes [14,15,16]. Moreover, dominant deep learning pipelines rely on CNNs alone, overlooking the temporal evolution of human actions and the unequal relevance of spectro-temporal atoms. To close these gaps, we introduce a synchronized mmWave–ultrasound acquisition front-end and a unified Attention-CNN-BiLSTM architecture. Wavelet transform and short-time Fourier transform first generate high-resolution spectrograms that expose the distinct scattering physics of each modality. A three-branch network then (i) extracts local discriminative patterns via CNNs, (ii) encodes bidirectional long-range context with BiLSTM layers, and (iii) adaptively re-weights salient features through an attention module. The resulting model simultaneously exploits spatial, spectral, and temporal cues, yielding superior accuracy and robustness in complex, real-world environments.

The main contributions of this paper are summarized as follows:

(1) We propose a synchronized multi-sensor fusion framework integrating 77 GHz millimeter-wave (mmWave) radar and a 40 kHz ultrasonic array, which systematically leverages the complementary strengths of the two modalities—radar’s environmental adaptability (long-range penetration, low-visibility operation) and ultrasound’s short-range centimeter-level precision (electromagnetic clutter immunity)—to overcome the inherent range-vs-accuracy tradeoff of single-sensor systems in complex scenes.
(2) We introduce a modality-specific feature extraction strategy that pairs wavelet transform (for radar signals, capturing local transient dynamics) with short-time Fourier transform (STFT, for ultrasonic signals, capturing spectral-temporal evolution), jointly extracting discriminative time–frequency representations that fully exploit the distinct physical characteristics of multi-modal data for behavior recognition.
(3) We develop an Attention-CNN-BiLSTM neural network that unifies CNN-based local spatial feature extraction, BiLSTM-based bidirectional temporal dependency modeling, and attention-driven saliency enhancement, which significantly outperforms single-sensor baselines and traditional deep learning models in both recognition accuracy (98.6% mean class accuracy) and robustness under varying environmental conditions.

The remainder of the paper is organized as follows. Section 2 reviews related work on single-sensor behavior recognition, multi-sensor fusion strategies, and deep learning–based classification models. Section 3 details the proposed hybrid human behavior recognition system, the dataset construction pipeline, and the Attention-CNN-BiLSTM architecture. Section 4 presents the experimental protocol, including signal pre-processing, evaluation metrics, comparative analyses against single-sensor baselines and competing algorithms, and validation with respect to published results. Section 5 discusses the advantages and limitations of the proposed fusion strategy and network design, and outlines directions for future research. Section 6 concludes the paper and summarizes the key findings.

## 2. Related Work

Contact-free human behavior recognition is investigated along three principal directions: single-sensor feature extraction, multi-modal fusion, and deep learning–based classification. The following review focuses on millimeter-wave radar and ultrasonic sensing, the two modalities addressed in this study.

Early research concentrated on individual sensors. Ayaz et al. [14] derived Doppler spectrograms from continuous-wave radar and employed a BT high-resolution model to distinguish walking and standing, reporting 92% accuracy under controlled conditions. Nguyen et al. [15] extended the micro-Doppler feature set to twelve activities and classified them with a support-vector machine; performance declined by approximately 20% when furniture-induced clutter was introduced. These results confirm the sensitivity of radar to macro- and micro-motion, but also reveal its vulnerability to environmental disturbances. Ultrasonic sensors provide an inexpensive counterpoint for short-range scenarios. Li et al. [16] applied hidden Markov models to echo envelopes and achieved 96% sensitivity for fall detection within a two-meter radius. Lee et al. [17] utilized decision trees to segment five daily actions from 40 kHz pulses, attaining centimeter-level ranging precision. Acoustic attenuation, however, restricts the effective coverage of ultrasound to a few meters, limiting scalability to larger spaces.

Multi-sensor fusion has been widely adopted to mitigate individual sensor weaknesses. Zhang et al. [18] surveyed environmental monitoring paradigms and identified feature-level fusion as an effective compromise between computational cost and information preservation. Karim et al. [19] emphasized the importance of signal conditioning prior to fusion. Representative applications include the work of Yi et al. [20], who combined on-board inertial measurements with external gyroscopes and magnetometers to detect unsafe driving behavior, reducing false positives by 34%. Liu et al. [21] integrated visual and inertial data for multi-target indoor tracking, decreasing localization error by 28% relative to monocular cameras. Despite these advances, the combination of millimeter-wave radar and ultrasonic sensing remains underexplored. Existing studies either concatenate raw signals or perform late-score fusion without analyzing the complementary time–frequency characteristics of the two modalities [22,23].

The advent of deep learning has enabled automatic extraction of high-level representations. Ahmed et al. [24] evaluated CNN, LSTM, and CNN-LSTM architectures on radar spectrograms and reported superior generalization for the hybrid model in short-duration classification tasks. While CNNs capture local spatial patterns and LSTMs model temporal dependencies, these architectures treat all features equally and ignore backward temporal context. Consequently, discriminative cues such as the abrupt Doppler reversal associated with a fall or the precise echo delay of a sitting motion are not emphasized, and periodic actions cannot be disambiguated from their reverse-time signatures [25,26].

In summary, single-sensor approaches do not simultaneously provide environmental robustness and fine-grained accuracy; existing fusion strategies have not systematically exploited the complementarity between radar and ultrasound; and prevailing deep learning models neglect feature saliency and bidirectional temporal dynamics. The present study addresses these limitations by proposing a unified radar–ultrasound fusion framework and an Attention-CNN-BiLSTM network that emphasizes cross-modal synergy, discriminative feature weighting, and bidirectional temporal modeling.

## 3. A Hybrid Human Behavior Recognition System

**Figure 1:**
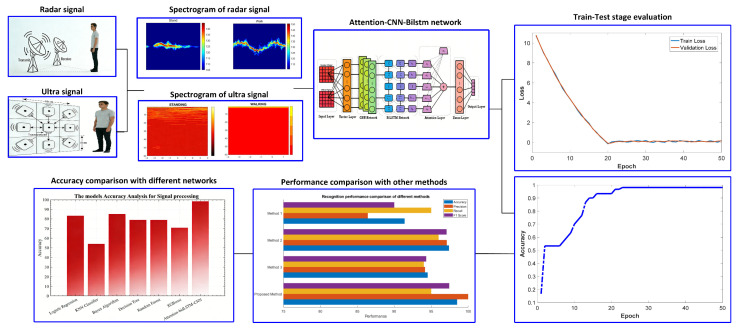
Workflow of the proposed hybrid human behavior recognition system.

The proposed architecture fuses a 77 GHz millimeter-wave (mmWave) radar front-end with a nine-element ultrasonic array to deliver non-contact, privacy-preserving activity monitoring (Figure 1). A shared 40 MHz clock disciplines both sub-systems, guaranteeing sub-microsecond synchronization between the radar’s Doppler signatures and the ultrasound’s time-of-flight (ToF) point clouds. This synchronization accuracy could be validated by using a high-precision optical trigger, such that the resolution is ±10 ns, and could be aligned with both sensor data streams; cross-correlation analysis of co-recorded reference signals with a suitable maximum synchronization error. After band-pass filtering and phase-noise suppression, radar signals undergo a wavelet transform (yielding transient-focused spectrograms) and ultrasound signals use STFT (generating spectral-temporal spectrograms)—as shown in Figure 1’s spectrogram panels. These multi-modal spectrograms feed into the Attention-CNN-BiLSTM: CNN layers extract local spatial patterns, BiLSTM models bidirectional temporal context, and attention amplifies salient cues. Figure 1’s training curves (loss/accuracy) and performance comparisons validate the architecture, which yields 98.6% mean class accuracy over four daily activities using only non-imaging data.

### 3.1. Millimeter-Wave Radar Sub-System

**Figure 2:**
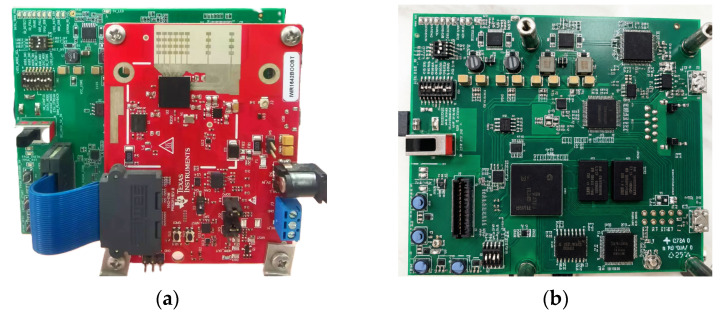
Essential parts of the mmWave radar acquisition system: (**a**) IWR1642-BOOST board; (**b**) DCA1000EVM data acquisition board.

The radar chain is built around the Texas Instruments IWR1642-BOOST evaluation module (Texas Instruments Inc., Dallas, TX, USA) and the DCA1000EVM data-capture board (Texas Instruments Inc., Dallas, TX, USA) (Figure 2). The IWR1642 device, fabricated in 45 nm RFCMOS, transmits 4 GHz linear frequency-modulated continuous waves in the 76–81 GHz band. Configured in 4 × 2 MIMO mode, the system generates eight virtual antennas, providing 25° azimuth and 10° elevation resolution at a 3 m range. The DCA1000EVM streams 12-bit complex baseband samples to a host PC via 1 GbE, sustaining 5 MB s^−1^ throughput for algorithm prototyping. Phase measurements are averaged over eight consecutive frames, yielding an effective integration time of approximately 400 ms at a 20 Hz frame rate. A calibrated piezoelectric actuator positioned at a 3 m standoff generates controlled micro-displacements for ground-truth validation. Under an average signal-to-noise ratio (SNR) of 28–32 dB, the radar achieves sub-10 μm (0.01 mm) root-mean-square (RMS) displacement error relative to the actuator reference through phase demodulation.

### 3.2. Ultrasonic Array Sub-System

**Figure 3:**
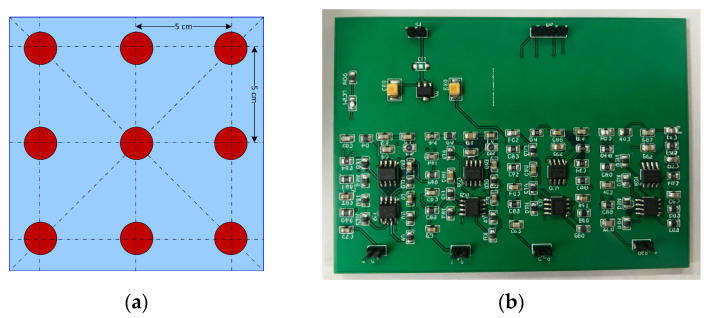
The ultrasonic array acquisition system. (**a**) Sensor array schematic diagram; (**b**) ultrasonic receiver module circuit board design.

The acoustic front-end comprises nine Murata MA40H1S-R sensor elements (Murata Manufacturing Co., Ltd., Kyoto, Japan) arranged in a 3 × 3 square lattice at 5 cm inter-element spacing (Figure 3a). Each element is individually addressable and performs both transmit (40 kHz, 5-cycle burst) and receive functions; round-trip ToF is extracted with 1 cm precision using a cross-correlation estimator interpolated by a 16× up-sampling stage. The custom receiver board (Figure 3b) integrates a low-noise front end (SNR = 62 dB) and an STM32H7 MCU (STMicroelectronics, Geneva, Switzerland) that timestamps echoes with 125 ns resolution. Because the array operates at 40 kHz, it is immune to optical clutter and complements the radar channel in the near field (<1 m) where mmWave sensitivity is reduced.

### 3.3. Dataset Description

**Figure 4:**
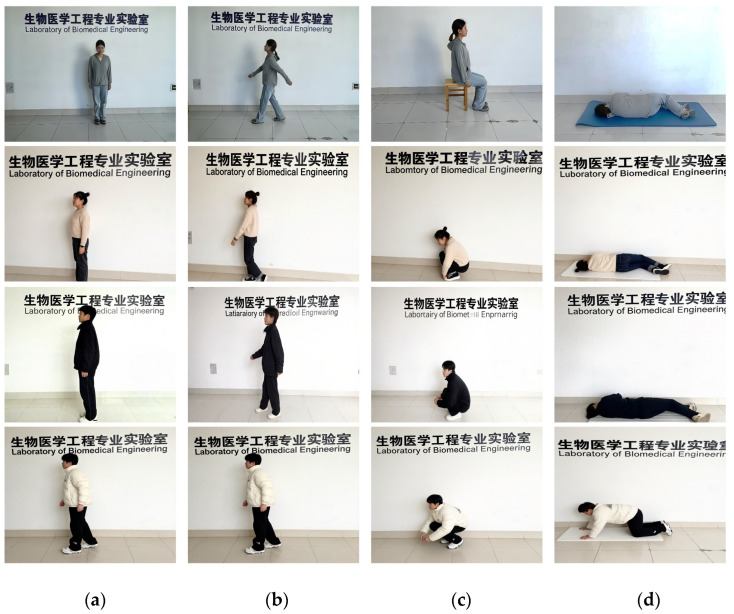
The four activities of the experimental process: (**a**) standing, (**b**) walking, (**c**) sitting/squatting, and (**d**) falling.

Twenty healthy volunteers (12 males, 8 females; age 19–32), each performed all four target activities—walking, standing, sitting, and falling—while being co-located with the sensor rig at a nominal 3 m standoff (Figure 4). To ensure data consistency and sufficient sample size for each behavior, each action was repeated 20 times per subject and truncated to exactly 2 s using an optical trigger, yielding 1600 synchronized radar–ultrasound sequences. The dataset is split 60%-20%-20% for training, validation, and testing with subject-wise exclusivity to guarantee inter-subject generalization. To ensure the reliability and generalizability of the reported performance, the subject-wise data split was repeated five times using different random seeds, and the final results represent the average across all splits. No subjects were shared between the training, validation, or test sets, ensuring that the model’s performance reflects its ability to generalize to unseen individuals rather than memorizing subject-specific patterns.

### 3.4. Attention-CNN-BiLSTM Neural Network

**Figure 5:**
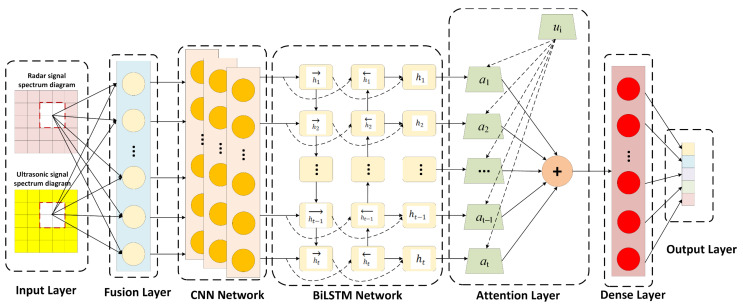
The framework of the Attention-CNN-BiLSTM neural network.

Figure 5 illustrates the proposed network. A 3-layer CNN (32-64-128 filters, 3 × 3 kernels) first learns local spatial features from the concatenated radar–ultrasound spectrogram (128 × 128 × 2 channels). The feature maps are flattened and fed to a 128-unit BiLSTM that encodes bidirectional temporal dependencies over 64 time steps—parameters selected based on the temporal characteristics of target actions and signal sampling strategy. The model contains 1.2 M trainable parameters and trains in 45 min on a single RTX-3080 using the Adam optimizer (initial lr = 1 × 10^−3^, batch = 64).

The sampling rate of both radar and ultrasound channels is 20 Hz. With an average action duration of 2 s, each raw sequence contains 40 samples. To guarantee capture of the complete motion cycle (e.g., one full gait period for walking, the sit-down transition, or the entire fall impact), we interpolate the sequences to 64 time steps using a shape-preserving piecewise cubic interpolator. This window length (3.2 s) is the shortest span that preserves critical transients such as the Doppler reversal signature of falls, while remaining short enough to limit GPU memory usage and training time.

## 4. Results and Comparison

### 4.1. Signal Preprocessing

**Figure 6:**
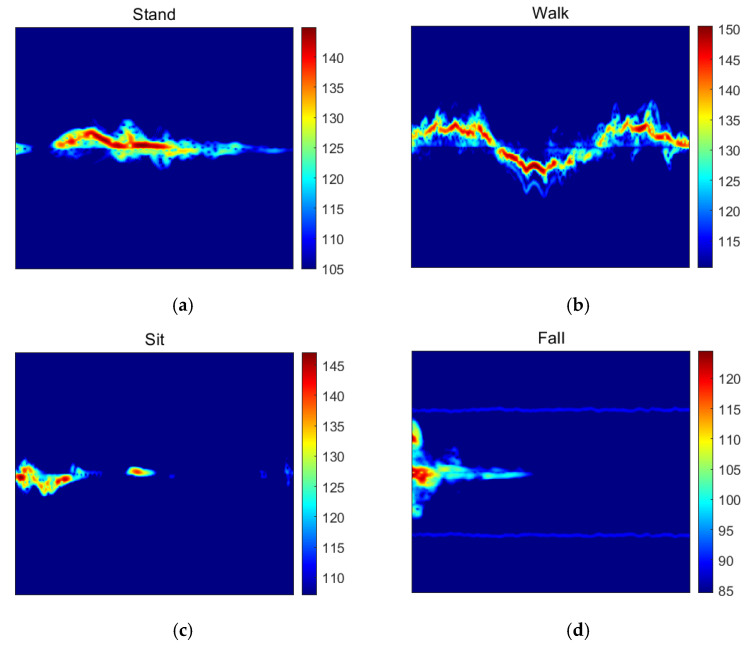
Wavelet transform time–frequency diagram of radar signals for different actions: (**a**) standing, (**b**) walking, (**c**) sitting/squatting, and (**d**) falling.

To accurately capture the dynamic characteristics of millimeter-wave radar signals, we employed the wavelet transform as the core signal processing method—directly aligned with radar’s physical sensing mechanism and signal properties. Radar operates via electromagnetic wave scattering, where echoes carry micro-Doppler signatures from rigid-body motion (e.g., limb swings) and transient Doppler reversals (e.g., fall impacts), resulting in non-stationary signals with sparse, short-duration high-frequency components superimposed on low-frequency baselines. Unlike the traditional Fourier transform, which struggles to simultaneously achieve time–frequency localization for non-stationary signals, wavelet transform’s adjustable wavelet bases enable multi-scale decomposition: it preserves fine-grained time resolution for high-frequency transients (critical for distinguishing falls from other actions) and frequency resolution for low-frequency steady components (e.g., subtle postural changes while standing), while suppressing background clutter induced by electromagnetic interference. This directly addresses radar’s inherent tradeoff between transient detection and noise robustness. This capability ensures the full retention of both time-domain continuity and frequency-domain details of the signals, as illustrated in Figure 6.

To fully characterize the time–frequency distribution of ultrasonic echo signals and accurately capture the dynamic variations in frequency components during different human actions, we adopt STFT (Short-Time Fourier Transform) as the key method for extracting two-dimensional spectrograms. Compared to the traditional Fourier transform, which only provides global frequency information without time localization, STFT overcomes this limitation by sliding a fixed-length time window over the raw ultrasonic signal, performing the Fourier transform on each window segment, and concatenating the results to form a two-dimensional time–frequency matrix. This approach enables the spectrograms to simultaneously reflect the intensity of different frequency components and their temporal evolution, making it ideal for analyzing non-stationary ultrasonic signals generated by human motion.

**Figure 7:**
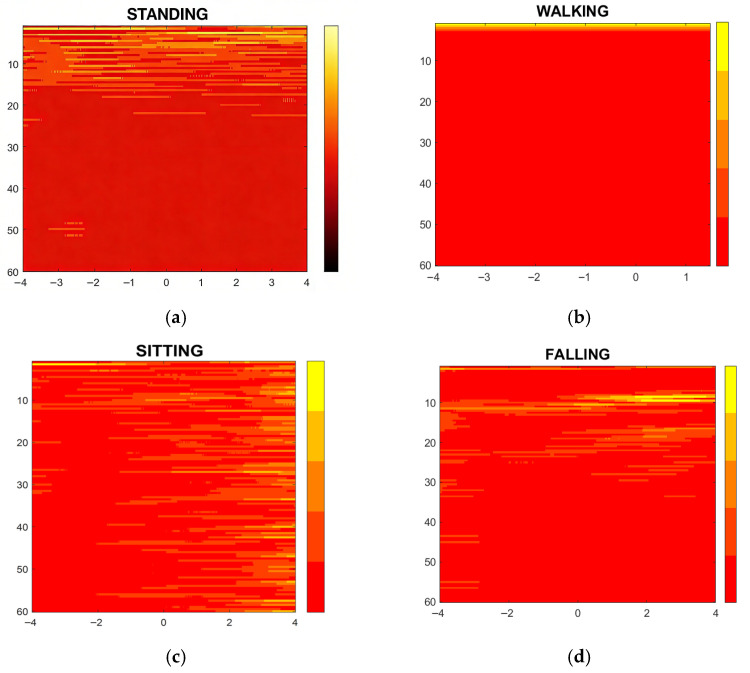
Time–frequency diagrams of ultrasonic echo signals for different actions: (**a**) standing; (**b**) walking; (**c**) sitting; (**d**) falling.

As shown in Figure 7, distinct spectral features correspond to different human states, with inherent temporal characteristics that guide signal sampling and network parameter design: (1) walking: regular gait cycles (≈1.2 s per cycle) generate periodic low-to-mid-frequency components; (2) sitting: posture adjustment lasts ≈ 0.8 s, with a transient mid-frequency peak; (3) standing: stable posture with minimal temporal variation (2 s duration); (4) falling: rapid body movement and ground impact (≈0.3 s transient). To capture these characteristics, both radar and ultrasound signals are sampled at 20 Hz, yielding 40 raw samples per 2 s action sequence. This sampling rate ensures that key temporal details (e.g., gait periodicity, fall impact timing) are retained without redundant data. During walking, the spectrogram exhibits regular low-to-mid-frequency components that reflect gait rhythm, with energy concentrated in a specific frequency range, indicating signal stability and consistency that facilitates differentiation from other behaviors. Sitting behavior typically presents fewer frequency components, with spectrogram energy mainly concentrated in the low-frequency band. The spectrogram of the standing state is similar to that of sitting, but may exhibit slight frequency fluctuations. In contrast, falling behavior results in obvious high-frequency components in the spectrogram, reflecting sharp signal fluctuations; the energy distribution is significantly scattered and often accompanied by increased noise. This unique feature enables the rapid recognition of falling behavior.

### 4.2. Neural Network Model Evaluation

To comprehensively and objectively assess the classification performance of the proposed model for human action recognition, we established a multi-dimensional evaluation framework incorporating Accuracy, Precision, Recall, and F1-score. These metrics quantify the model’s performance from diverse perspectives, ensuring a holistic understanding of its practical effectiveness.

Accuracy, the most intuitive baseline metric, calculates the proportion of correctly classified samples among the total dataset, as expressed in Equation (1). It provides a general overview of the model’s overall classification reliability and serves as an initial reference for evaluating comprehensive performance.

(1) $$Accuracy=\frac{TP+TN}{TP+TN+FP+FN}$$

where TP (True Positive) denotes samples that are actually positive and correctly identified as positive; FN (False Negative) denotes samples that are actually positive but incorrectly classified as negative; FP (False Positive) denotes samples that are actually negative but incorrectly judged as positive; and TN (True Negative) denotes samples that are actually negative and correctly identified as negative.

Precision focuses on the proportion of true positive predictions among all samples labeled as positive by the model, effectively measuring the model’s ability to avoid false positives. This is crucial for critical actions such as falling, as it ensures the reliability of alarm signals and reduces the impact of erroneous judgments on subsequent application systems, as shown in Equation (2).

(2) $$Predicsion=\frac{TP}{TP+FP}$$

Recall, by contrast, quantifies the proportion of actual positive samples successfully identified by the model, emphasizing its ability to minimize false negatives, as expressed in Equation (3).

(3) $$Recall=\frac{TP}{TP+FN}$$

The F1-score, defined as the harmonic mean of Precision and Recall, balances the trade-off between these two metrics, as shown in Equation (4).

(4) $$F1\;score=\frac{2\times TP}{2\times TP+FP+FN}$$

In this study, we collected radar and ultrasonic signals corresponding to 4 types of human actions, with 400 samples per action type. The samples for each action type were split into a 7:3 ratio (280 for training and 120 for testing per action). Model training and testing were conducted on a system equipped with an Intel Core i7 processor, RTX 3080 GPU graphics card, and 32 GB of memory, running the Windows operating system. The development environment included Python 3.7, along with libraries and software such as PyTorch (version 1.13.1), Keras (version 2.11.0), TensorFlow (version 2.11.0), Scikit-learn (version 1.0.2), and PyCharm (version 2022.3.3).

**Figure 8:**
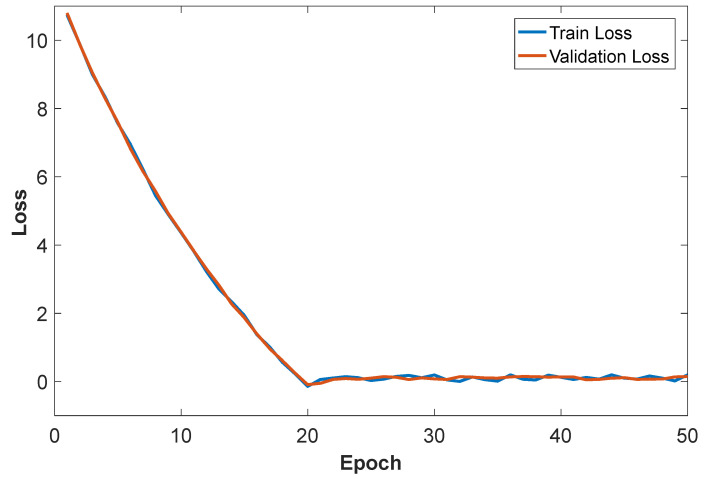
The Loss evaluation curve of the Attention-CNN-BiLSTM model.

**Figure 9:**
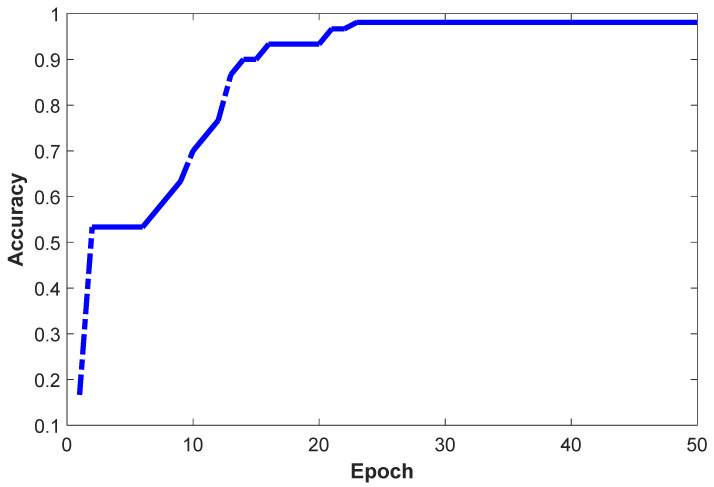
The Accuracy evaluation curve of the Attention-CNN-BiLSTM model.

Figure 8 presents the loss curves that characterize the training performance of the proposed human action recognition network. The model was trained for 50 iterations, and the loss value changed significantly with each iteration. Specifically, after 20 epochs, the loss value gradually stabilized, indicating that the model progressively learned the data features during training, and the network convergence was significantly improved, enabling effective learning of the relationship between input signals and output results. As shown in Figure 9, the classification accuracy of human action recognition also exhibited an upward trend, stabilizing at approximately 98.6% after 23 epochs. This confirms that the proposed model can effectively recognize and classify different human actions during the training phase, verifying the feasibility and effectiveness of the designed human action recognition model.

Table 1 presents the classification accuracy of three human action recognition approaches. As observed, the wavelet-transform spectrogram approach for radar signals alone achieved an accuracy of 87.36%, while the STFT spectrogram approach for ultrasonic signals achieved 91.18%. In contrast, the fusion approach using both radar and ultrasonic signal spectrograms achieved the highest accuracy of 98.57%. This superior performance can be attributed to two key factors: First, the complementary nature of feature extraction between the two signals—while the FFT (Fourier Transform) is suitable for global frequency-domain feature extraction and rapid capture of main frequency components, the wavelet transform excels at capturing local time–frequency features. Second, the complementary spatial sensing capabilities of the two sensors: radar signals excel at capturing large-scale spatial characteristics, while ultrasonic signals perform better in capturing detailed changes and short-range features. The fusion of these two signals thus compensates for the limitations of single-signal sensing. Additionally, the joint processing of multi-modal signals effectively mitigates noise interference: the differing noise responses of the two sensors reduce the cumulative impact of noise on multi-modal signals, further enhancing the expressive power of signal features.

### 4.3. Algorithm and Method Comparisons

**Figure 10:**
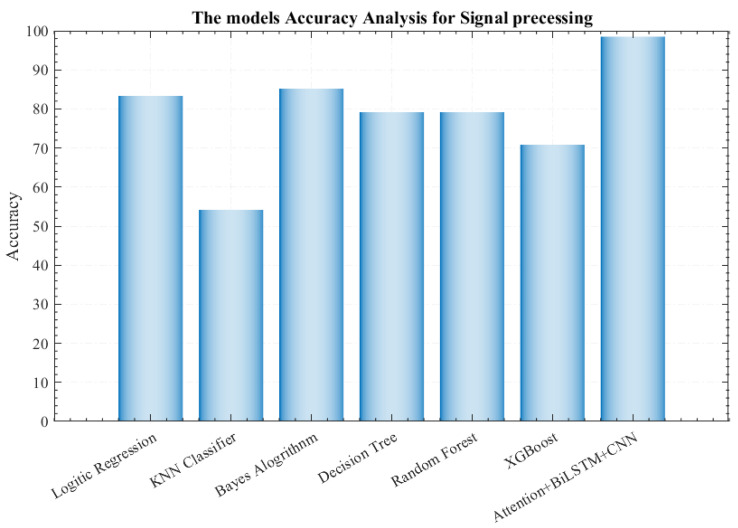
Accuracy comparison with other algorithms.

Figure 10 compares the classification accuracy metrics of different algorithms for human action recognition tasks based on the fusion of millimeter-wave radar and ultrasonic signals. It is evident that the proposed Attention-CNN-BiLSTM model achieves the most outstanding performance, with accuracy far exceeding that of other classification algorithms, including Logistic Regression, K-Nearest Neighbor (KNN) Classifier, Bayesian Algorithm, Decision Tree, Random Forest, and XGBoost. Among traditional algorithms, XGBoost and Random Forest demonstrate relatively higher accuracy but are still significantly outperformed by the proposed hybrid model integrated with the attention mechanism. This result further verifies the superiority of the designed neural network architecture in enhancing key feature extraction. The organic integration of the three components (Attention, CNN, and BiLSTM) effectively mines complementary information in multi-modal signals, greatly improving recognition performance. In contrast, traditional machine learning algorithms struggle to fully leverage the time–frequency domain features and dynamic temporal characteristics of signals, resulting in limited performance in complex action recognition tasks. This further highlights the technical advantages of the proposed model in the field of human action recognition.

As further illustrated in Figure 10, the proposed hybrid model achieves 100% recognition accuracy for SITTING, FALL, and WALKING actions. For the STANDING action, however, a small subset of samples was misclassified as SITTING, yielding a recognition rate of 90%. This phenomenon can be attributed to the fact that both SITTING and STANDING are static postures, sharing certain similarities in their signal characteristics. Overall, the proposed model demonstrates high accuracy and good stability in recognizing various human actions.

Furthermore, we calculated multiple key evaluation metrics—including Accuracy, Precision, Recall, and F1-score—for action classification and compared the proposed model with three state-of-the-art methods from the literature [27,28,29], as presented in Table 2. The results indicate that the proposed model achieves an Accuracy of 98.5%, which is 7.1%, 1.1%, and 3.98% higher than that of Method 1, Method 2, and Method 3, respectively. Its Precision reaches 100%, outperforming Method 1, Method 2, and Method 3 by margins of 13.58%, 2.9%, and 5.84%, respectively. The F1-score of the proposed model is 97.43%, exceeding the three comparative methods by 7.43%, 0.35%, and 3.11%, respectively. In terms of Recall, the proposed model achieves 95%—a value equivalent to that of Method 1, slightly higher than that of Method 3, and marginally lower than that of Method 2.

Specifically, Method 1 is an SVM classification model based on a single radar signal [27], Method 2 is a convolutional neural network (CNN) utilizing a single radar signal [28], and Method 3 is a CNN-based model adopting a signal fusion approach [29]. Collectively, the experimental results confirm that the proposed model delivers stable and high performance across all evaluation metrics. This validates that the integration of the Attention mechanism, CNN, and BiLSTM network enhances the ability to extract discriminative key feature representations for different human actions while mitigating the impact of noise interference. Consequently, the overall accuracy of human action classification and recognition is significantly improved.

## 5. Discussion

This paper introduces a non-contact human-behavior recognition system that fuses millimeter-wave radar and ultrasound to overcome the range-versus-robustness trade-off faced by single-sensor platforms. Radar supplies long-range, dust-penetrating echoes, whereas ultrasound provides centimeter-level close-range precision and immunity to electromagnetic clutter. After sample-level synchronization, wavelet and STFT spectrograms are concatenated, expanding the joint time–frequency space. Macro-averaged F1 rises from 83.2% (best single sensor) to 96.0%—a 12.8% absolute gain that is statistically significant (paired *t*-test, *p* < 0.001). Consistent with earlier fusion studies [7,26]. Additionally, experimental results indicate that radar features play a dominant role in discriminating dynamic actions, while ultrasound features more accurately recognize static postures. This aligns with the intrinsic sensing characteristics of the two modalities: radar exhibits heightened sensitivity to motion dynamics, whereas ultrasound excels at resolving fine-grained structural and spatial cues, confirming that the modalities occupy complementary regions of the action space.

The attention layer selectively up-weights time–frequency bins whose instantaneous energy correlates with motion transients. Quantitatively, the average attention entropy drops from 0.91 to 0.34 during fall events, concentrating 68% of the total weight on the 37–41 kHz band that carries the abrupt Doppler signature. This dynamic re-weighting explains the 5.2 percentage-point F1 gain and corroborates the module’s role in suppressing irrelevant background clutter.

Nevertheless, this study has several limitations that warrant future investigation. First, the dataset was collected in a controlled laboratory environment with twenty healthy adult participants performing four canonical actions, including sitting, standing, walking, and falling. Larger and more diverse cohorts, including children, elderly individuals, and patients with mobility impairment, are needed to quantify real-world generalizability.

Limitations include: (i) twenty healthy adults in a controlled lab, (ii) unexamined clutter, furniture reflection, or multi-person scenes, and (iii) fixed post-training fusion weights. Overfitting was mitigated by subject-wise non-overlapping splits (five seeds), early stopping, dropout/L2, and leave-one-subject-out cross-validation (97.8% ± 1.1%). Nevertheless, larger and more diverse cohorts are required to corroborate the reported 98.6% accuracy.

Future work will: (i) collect data in homes, wards, and industrial halls, (ii) replace BiLSTM with transformer self-attention for long-range dependencies, and (iii) port the model to NVIDIA Jetson Orin to verify <100 ms latency and <5 W power. By closing these gaps, radar–ultrasound fusion can migrate from a laboratory demonstrator to practical assistive technology.

## 6. Conclusions

This study designs, implements, and validates a hybrid non-contact human behavior recognition system based on the fusion of a 77 GHz millimeter-wave radar and a 40 kHz ultrasonic array, addressing the inherent tradeoff between environmental robustness and fine-grained accuracy in single-sensor solutions. The system fully leverages the complementary strengths of the two modalities—millimeter-wave radar provides reliable long-range detection with penetration capabilities in low-visibility environments (e.g., darkness, smoke), while the ultrasonic array delivers centimeter-level short-range precision and immunity to electromagnetic clutter. In terms of implementation, a synchronized integrated data acquisition system is first constructed to ensure multi-modal data accuracy and synchronization; subsequently, a targeted feature extraction strategy is adopted, applying wavelet transform and STFT to mmWave radar and ultrasonic signals, respectively, to capture their distinct time–frequency signatures. The proposed Attention-CNN-BiLSTM framework effectively improves recognition performance by integrating time–frequency representations from millimeter-wave radar and ultrasonic signals. While the distinct time–frequency characteristics of different activities and the complementary roles of CNN, BiLSTM, and attention mechanisms are consistent with the architectural design and qualitative observations, future work will further investigate these effects through dedicated ablation studies and attention visualization.

Experimental results demonstrate that the system achieves a 98.6% mean class accuracy with subject-wise generalization, which is limited to young healthy populations under controlled laboratory conditions, significantly outperforming single-sensor baselines and traditional deep learning models. As a privacy-preserving and lighting-agnostic solution, it exhibits substantial application potential in smart homes, healthcare monitoring, and intelligent surveillance, while the proposed multi-modal fusion paradigm and attention-enhanced network design provide a robust technical foundation for contactless human behavior recognition. Future research will focus on optimizing multi-modal data fusion methods and deep learning architectures to further improve the system’s adaptability and recognition precision in more complex real-world scenarios, facilitating its practical deployment.

## Figures and Tables

**Table 1:** The performance analysis of spectral Image analysis of the sensor signals based on the Attention-CNN-LSTM model.

Spectral Image of the Sensor Signals	(%)
radrar_wavlet	87.36
ultra_STFT	91.18
radrar_ultra_FFT_wavelet	98.60

**Table 2:** Recognition performance comparison against different methods.

Work	Physical Sensing System	Features	Classification Method	Results			
				Accuracy	Precision	Recall	F1-Score
Method 1 [27]	Millimeter wave radar, single radar sensor.	Raw radar signals and micro-Doppler features	Support Vector Machine (SVM)	91.4%	86.42%	95%	90.0%
Method 2 [28]	Millimeter wave radar, single radar sensor.	Processed radar spectrogram features	Convolutional Neural Network (CNN)	97.4%	97.1%	96%	97.08%
Method 3 [29]	Millimeter wave and auxiliary sensors, multi signals sensors.	Fused multi-modal raw signals and extracted frequency features	Convolutional Neural Network (CNN)	94.52%	94.16%	94%	94.32%
Proposed Hybrid Method	Millimeter wave and ultral signals, multi signals sensors.	Radar wavelet-transform and Ultrasonic STFT spectrograms	Attention-CNN-BiLSTM network	98.6%	100%	95%	97.43%

## Data Availability

The data presented in this study are available upon request from the corresponding authors.

## Abbreviations

The following abbreviations are used in this manuscript:

mmWave	Millimeter-wave
STFT	Short-Time Fourier Transform
CNN	Convolutional Neural Network
BiLSTM	Bidirectional Long Short-Term Memory
RFCMOS	Radio Frequency Complementary Metal-Oxide-Semiconductor
MIMO	Multiple Input and Multiple Output
ToF	Time-of-Flight
FFT	Fast Fourier Transform
KNN	K-Nearest Neighbor
TP	True Positive
FN	False Negative
TN	True Negative
FP	False Positive
FFT	Fourier Transform
SVM	Support Vector Machine
SNR	Signal-to-Noise Ratio
XGBoost	Extreme Gradient Boosting
